# The T2T genome assembly of *Ziziphus jujuba* ‘Huizao’ and pan-genome analyses provide insights into fruit texture diversity in jujube

**DOI:** 10.1186/s43897-025-00228-1

**Published:** 2026-06-05

**Authors:** Juan Jin, Aidi Zhang, Bingqi Shen, Xingnuo Li, Jacob B. Landis, Chong Chen, Ye Yuan, Lili Li, Lei Yang, Siyu Qu, Yalan Li, Xiujun Zhang, Mengjun Liu, Yanxia Sun, Dingyu Fan, Qing Hao

**Affiliations:** 1 Key Laboratory of Genome Research and Genetic Improvement of Xinjiang Characteristic Fruits and Vegetables, Institute of Fruits and Vegetables, Xinjiang Academy of Agricultural Sciences, Urumqi, 830091 China; 2 State Key Laboratory of Plant Diversity and Specialty Crops, Wuhan Botanical Garden, Chinese Academy of Sciences, Wuhan, 430074 China; 3 School of Integrative Plant Science, Section of Plant Biology and the L.H. Bailey Hortorium, Cornell University, Ithaca, NY 14853 USA; 4 College of Forestry and Landscape Architecture, Xinjiang Agricultural University, Urumqi, 830052 China; 5 School of Mathematics and Statistics, Wuhan University of Technology, Wuhan, 430070 China; 6 College of Horticulture, Hebei Agricultural University, Baoding, 071001 Hebei China

*Ziziphus jujuba* Mill., commonly known as jujube (2n = 2x = 24), is the most economically important fruit tree in the Rhamnaceae family (Zhang et al. 2011). Particularly, *Z. jujuba* is well known for its strong tolerance to drought and salt stress and the high economic and medical value of its fruit (Liu et al. 2020; Rashwan et al. 2020). Fruit texture which determines the taste and storage of jujube fruits is one main driver of consumer purchasing decisions (Song et al. 2023). Based on the texture of ripe fruits, jujube cultivars are categorized into fresh-eating (e.g., 'Dongzao') and dry-eating types (e.g., 'Huizao'). Fresh-eating jujubes have a crisp yet tender texture, whereas dry-eating jujubes are firmer and less crisp. Pectin is a major component of the primary cell wall and middle lamella. It maintains the physical stability and mechanical strength of the cell wall, playing a crucial role in determining fruit texture (Chen et al. 2024). Studies have also shown that pectin fragments can activate WAKs (wall associated kinases) via binding to the GUB_WAK_bind domain, triggering cell wall biosynthesis and increasing cell wall thickness—a key factor in fruit firmness (Cai et al. 2023). To date, little is known about GUB_WAK_bind genes and their relationship with fruit texture diversity in jujube.

In this study, we generated a telomere-to-telomere (T2T) haplotype-resolved genome assembly for 'Huizao', integrating 30.42 Gb of HiFi CCS reads, 36.63 Gb of ONT reads, and 50.18 Gb of Hi-C data (Tables S1-S3; Figs. 1A, S1). Genome integrity assessment revealed BUSCO completeness scores of 98.2% (HZ_hapA) and 98.3% (HZ_hapB) (Table S4). Illumina short-read and HiFi long-read mapping rates reached 99.39% and 99.36% for HZ_hapA and HZ_hapB, respectively. Long terminal repeat (LTR) assembly index (LAI) analysis demonstrated values of 20.34 (HZ_hapA) and 20.46 (HZ_hapB). Telomeric repeats were identified on all 24 chromosomes in both haplotypes, except at the 5′ end of chromosome 6 in HZ_hapB. Putative centromeric regions were annotated on all chromosomes except chromosome 3 in HZ_hapB (Table S5). About half of the assembled genome sequences were annotated as repeat elements (Table S6). Finally, 30,369 and 30,005 protein-coding genes were predicted and annotated in HZ_hapA and HZ_hapB, respectively (Tables S7, S8).

**Fig. 1:**
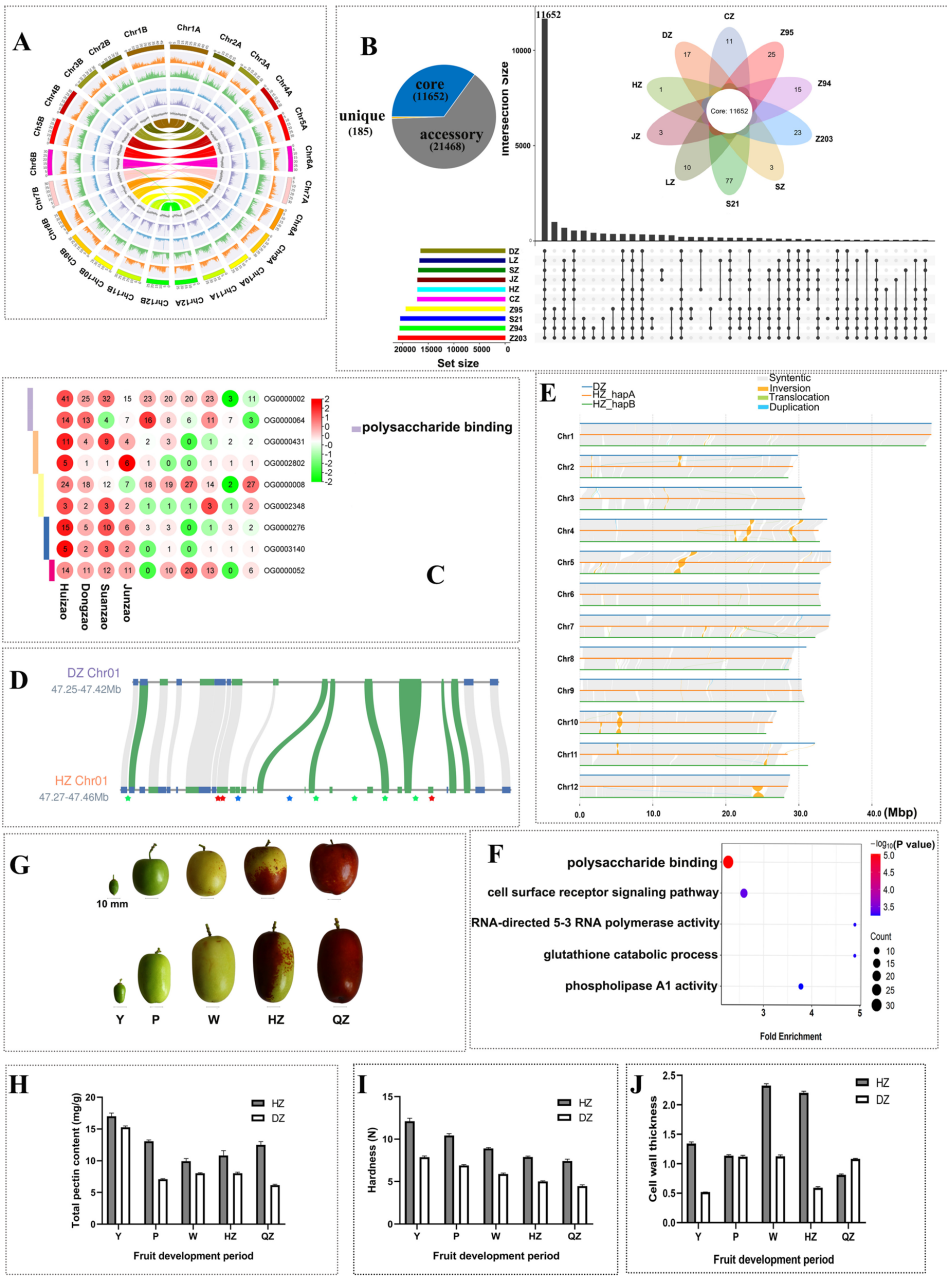
**A** Circos plot of *Z. jujuba* 'Huizao' haplotype-resolved gap-free genomic features. From the outermost track to the innermost track of the genome physical map, the components are arranged in order as follows: Ideograms of chromosomes, Gene density, LTR density, Copia type LTR density, Gypsy type LTR density, GC content density, Collinear relationship between the two haplotypes of 'Huizao' genomes. All the density information was counted with 200-kb windows. **B** Upset Venn diagram of gene families among ten jujube genome assemblies. DZ, 'Dongzao'; HZ, 'Huizao'; JZ, 'Junzao'; SZ, 'Suanzao'; CZ, 'Lingwuchangzao'; LZ, 'Shiguang'. **C** Heatmap of expanded orthogroups in the 'Huizao' genome, the number in the circle represents gene number in the orthogroup, GO terms are highlighted by boxes with different colors. **D** Chromosome distribution of clade-a; the collinear links of expanded GUB_WAK_bind genes are highlighted with green colors, and the collinear links of other genes are shown with grey colors. Expanded GUB_WAK_bind genes in 'Huizao' are labeled with green stars; the adjacent transposons and chaperone genes are labeled with red and blue stars, respectively. **E** Synteny analysis of HZ_hapA, HZ_hapB, and DZ genome assemblies. **F** GO enrichment analysis of genes located within structural variation regions. **G** Fruit morphology and developmental stages (31, 63, 78, 98, and 108 days after flowering) in 'Huizao' and 'Dongzao', designated as young (Y), expansion (P), white ripening (W), half ripening (HZ), and full ripening (QZ). **H** Dynamics of pectin content during fruit development. **I** Dynamics of fruit firmness during fruit development. **J** Dynamics of cell wall thickness during fruit development

Collectively, the assembly achieved gold-standard quality, evidenced by exceptional BUSCO scores, near-perfect read mapping rates, high LAI values, and comprehensive annotation of telomeres, centromeres across nearly all chromosomes.

Using one 'Huizao' genome assembly and nine other published jujube genome assemblies, we further constructed a graph-based jujube pan-genome (Figs. 1B, S2). The final pan-genome size was 556 Mb, comprising nodes and edges (Table S9) and encoding 323,139 gene models. Ortholog clustering classified all genes from the ten assemblies into 25,575 gene families. Among these, 11,652 were core genes, 21,468 were dispensable genes, and 185 were private genes (Fig. 1B). The pan-genome analysis revealed extensive genomic diversity, with a substantial fraction of the gene repertoire being variable across cultivars, highlighting the dynamic nature of the jujube genome.

Phylogenetic analysis based on 1,718 single-copy genes classified the four jujube varieties with T2T genome assemblies into two clades (Fig. S3a). A total of 460 and 344 gene families were significantly (*P* < 0.01) overrepresented and underrepresented in 'Huizao', respectively. Ks value analysis revealed a shared peak around 0.001 across jujube varieties (Fig. S3b), and self-alignment of each variety confirmed no recent whole-genome duplication (WGD) events—only the ancestral γ triplication event was detected. Gene family comparison identified 22,577 gene families among the four varieties, with 86 unique to 'Huizao' (Fig. S3c). GO enrichment analysis showed that expanded gene families in 'Huizao' were primarily associated with polysaccharide binding, while its unique gene families were enriched in pectin metabolic processes (Figs. S3d, 3e).

We further examined gene copy number variation for the enriched GO term polysaccharide binding and identified two orthogroups (OG0000002, OG0000064) associated with this function. In OG0000002, 'Huizao' contained 41 gene copies, compared to 25 in 'Dongzao', 32 in 'Suanzao', and 15 in 'Junzao'. For OG0000064, 'Huizao' again had the highest copy number (Fig. 1C). Notably, all genes in both orthogroups harbored the conserved GUB_WAK_bind domain sequences. Given the sister relationship between 'Dongzao' and 'Huizao' revealed by phylogenetic analysis (Fig. S3a) and supported by Guo et al. (2024) , we identified all GUB_WAK_bind gene members in 'Huizao' (123 genes) and 'Dongzao' (92 genes). Phylogenetic analysis of these genes classified them into three subgroups exhibiting distinct domain architectures (Figs. S4a, S4c, S4d, S5, S6), with GUB_WAK_bind genes from OG0000002 and OG0000064 distributed across all subgroups. Chromosomal distribution analysis showed that these genes underwent tandem duplication and were primarily located on chromosomes 1, 4, and 10 (Figs. 1D, S4b). To further investigate the evolutionary dynamics, we analyzed the collinearity and flanking genes of GUB_WAK_bind genes in OG0000002 and OG0000064 in both 'Huizao' and 'Dongzao' (Figs. 1D, S4b, S7). Notably, many transposon-related genes were found adjacent to GUB_WAK_bind genes in 'Huizao' but were absent in 'Dongzao' (Figs. 1D, S4b), suggesting a transposon-driven expansion of this gene family in 'Huizao'.

Given the pronounced phenotypic differences between 'Huizao' and 'Dongzao', we performed a comprehensive genome comparison between the two varieties. This analysis identified 435,321 single nucleotide polymorphisms (SNPs), 51,245 insertions, 51,864 deletions, 36 inversions, 145 translocations, and 215 duplications (Fig. 1E, Table S10). Using 'Huizao'_hapA as the reference, we detected 6,752 structural variants (50–10,000 bp) in the 'Dongzao' genome (Fig. S8, Table S11). Further annotation of genes within these variant regions revealed that 3,400 genes were associated with 2,204 structural variants. GO enrichment analysis showed that these structural variant-related genes were also significantly enriched in polysaccharide-binding (Fig. 1F).

Ultramicroscopic structural analysis of cell pulp and physiological measurements revealed significant differences between 'Huizao' and 'Dongzao' across five fruit developmental stages (Fig. 1G). Compared to 'Dongzao', 'Huizao' fruits exhibited higher pectin content, greater firmness, and thicker cell walls (Figs. 1H-1J, S10). Furthermore, several GUB_WAK_bind genes showed differential expression between 'Huizao' and 'Dongzao', with most upregulated at the Y or P stage in 'Huizao' (Fig. S9a). We also identified numerous differentially expressed genes (DEGs) between the two cultivars at all five developmental stages (|FoldChange|> 1; *P* < 0.01) (Table S12). GO enrichment analysis indicated that these DEGs were associated with processes including cellular/cell-wall polysaccharide metabolism, cellular response to abscisic acid stimulus, cell wall thickness regulation, and symbiont-induced programmed cell death (Figs. S11-13). Examination of genomic variations near these DEGs (Table S12) revealed that most harbored diverse variants, including small indels (< 50 bp), SNPs, and large structural variants (SVs). Notably, many GUB_WAK_bind genes exhibited both differential expression and genomic variations (Figs. S8, S9b, S14).

In summary, we generated the first T2T haplotype-resolved genome assembly for 'Huizao' jujube and performed pan-genome and comparative genomic analyses. The high-quality 'Huizao' assembly and the jujube pan-genome provide a comprehensive genomic resource for future research and crop improvement in this perennial fruit tree. Notably, we identified and characterized the GUB_WAK_bind gene family. Our analysis revealed that divergence between the dry-eating 'Huizao' and fresh-eating 'Dongzao' cultivars involves not only GUB_WAK_bind gene copy number variation but also differences in expression levels and genomic sequence, effectively explaining their phenotypic differences. By integrating genomic, transcriptomic, and fruit development data, we propose a potential mechanistic model wherein WAKs encoded by GUB_WAK_bind genes respond to pectin-derived signals to modulate fruit cell wall thickness. These findings offer insights into the evolution of the GUB_WAK_bind family and the genetic basis underlying fruit texture diversity in jujube.

## Supplementary Information

Supplementary Material 1.

Supplementary Material 2.

Supplementary Material 3.

Supplementary Material 4.

## Data Availability

The genome assembly of 'Huizao' and raw reads produced by transcriptome sequencing in the current study have been submitted to the National Center for Biotechnology Information (NCBI) under BioProject PRJNA1262080.
