# Supplementary material for: The T2T genome assembly of Ziziphus jujuba ‘Huizao’ and pan-genome analyses provide insights into fruit texture diversity in jujube
Source: Mol Hortic. 2026 Jun 5;6:42. doi: 10.1186/s43897-025-00228-1 (PMC13238111; doi:10.1186/s43897-025-00228-1)
Supplement: Supplementary file 2 — Supplementary Material 2. [file 43897_2025_228_MOESM2_ESM.docx]

**Materials and methods**

**Plant materials**

Fresh leaf samples of *Z. jujuba* 'Huizao' for genome sequencing were collected in 2023 from the experimental base of the Fresh Jujube Production Demonstration Park in Yangtake Township, Maigaiti County, Xinjiang Uygur Autonomous Region (70.40° E, 38.54° N). Additionally, fruits at five developmental stages (31, 63, 78, 98, and 108 days after flowering, representing the young fruit, expansion, white ripening, half ripening, and full ripening stages, respectively) were sampled from both 'Huizao' and 'Dongzao' cultivars at the same location for RNA sequencing. All samples were immediately stored in liquid nitrogen until DNA/RNA extraction and sequencing.

**Genome sequencing**

**High-quality genomic DNA of 'Huizao' was extracted using a modified CTAB method (Murray and Thompson, 1980), followed by assessments of concentration, purity, and integrity. The quantified DNA was first subjected to Illumina sequencing: a 350 bp insert library was prepared with the TIANSeq DirectFast DNA Library Prep Kit (Illumina) and sequenced on the Illumina NovaSeq 6000 platform. Subsequently, a PacBio HiFi library was constructed using the Pacific SMRTbell Express Template Prep Kit 2.0 (Pacific Biosciences, USA) following the standard protocol and sequenced on the PacBio Sequel II platform. For Nanopore sequencing, the ONT library was prepared with the SQK-LSK109 kit (Oxford Nanopore) according to the manufacturer’s instructions and sequenced on a PromethION device. Finally, the Hi-C library was constructed using the NEBNext® Ultra™ II DNA Library Prep Kit for Illumina (NEB) and sequenced on the Illumina NovaSeq 6000 platform.**

**RNA-seq sequencing**

Total RNA was extracted from fruits of 'Huizao' and 'Dongzao' at five developmental stages, as described above, with three biological replicates per stage. RNA purity was assessed using a NanoPhotometer spectrophotometer (IMPLEN, CA, USA). Sequencing libraries were prepared with the TruSeq Stranded mRNA LT Sample Prep Kit (Illumina, San Diego, CA, USA) following the manufacturer’s protocol, and 150-bp paired-end sequencing was performed on the Illumina NovaSeq 6000 platform.

**Genome assembly, annotation and evaluation**

A hybrid assembly strategy combining HiFi reads and ONT reads was employed to generate the 'Huizao' genome contigs using HiFiasm (v0.18.2-r467) (Cheng et al., 2021). Subsequently, Hi-C reads were mapped to the assembled contigs with Juicer (v1.6) (Durand et al., 2016), and 3D-DNA (Dudchenko et al., 2017) was used to cluster and order them into chromosomes. To achieve a gap-free 'Huizao' genome assembly, ONT reads were further assembled into ultra-long contigs using NextDenovo, followed by alignment of these contigs to the chromosomal-level assembly with Winnowmap (Jain et al., 2020). This step enabled gap detection and filling, ultimately yielding a telomere-to-telomere (T2T) genome assembly.

We detected the telomeres in the gap-free genome assembly of 'Huizao' with the telomere identification tool tidk (Brown et al., 2023); the normalized unified sequence “TTTAGGG” in *Arabidopsis thaliana* was used to search terminal sequence in each chromosome. The centromere prediction was conducted with Trf (Benson, 1999) by searching the short tandem repeat elements in the genome (Chen et al., 2023; Song et al., 2021). According to the repeat length and copy numbers, elements were labeled and clustered to reduce redundancy; based on the total matching length and the content of duplicate components, continuous matching regions were scored and marked as centromeres.

For coding regions prediction, de novo prediction was performed using Augustus (v3.3.2) (Stanke et al., 2008), Genscan (v1.0) (Burge and Karlin, 1997) and GlimmerHMM (v3.0.4) (Majoros et al., 2004). The gene sets generated by the three methods were combined into high-confidence gene structure models with MAKER3 (v3.01.03) (Cantarel et al., 2008). The protein-coding genes were functionally annotated with Blastp (v2.0.11.148) (https://blast.ncbi.nlm.nih.gov/Blast.cgi?PROGRAM=blastp) against five public databases: SwissProt, NR, GO, KEGG and eggnog. The conserved domains and motifs were annotated with InterProScan (v5.61-93.0) (Zdobnov and Apweiler, 2001) by comparing with Pfam, PRINTS, PANTHER, ProSiteProfiles and SMART databases. Different types of ncRNAs were identified, the tRNA genes were identified using TRNASCAN-SE (v1.3.1) (Chan and Lowe, 2019), and the rRNA, miRNA and snRNA genes were identified by blasting against the Rfam (v13.0) database with Infernal (v1.2) (Nawrocki and Eddy, 2013).

To evaluate genomic consistency, the Illumina sequencing reads were mapped to the T2T genome assembly using BWA (v0.7.17-r1188) (Jung and Han, 2022). The genome completeness was assessed by BUSCO (v4.1.4) (Simao et al., 2015) using the ‘Eukaryota_odb10’ as reference. The genome assembly quality was assessed using the LTR assembly Index (LAI) by LTR_retriever (Ou and Jiang, 2018).

**Genome variation detection and pan-genome construction**

We collected published jujube genomes, including 'Junzao' (JZ), 'Suanzao' (SZ), 'Dongzao' (DZ), 'Jinsixiaozao' (Z94), 'Goutouzao' (Z203), 'Lingwuchangzao' (CZ), 'Shiguang' (LZ), S21, and Z95 (Guo et al., 2024; Li et al., 2024; Wei et al., 2024; Yang et al., 2023). The pan-genome graph based on ten jujube genome assemblies was constructed with the Minigraph-Cactus (v2.8.2) toolkit (Hickey et al., 2024), and visualized with ODGI (v2.6.1) (Guarracino et al., 2022). SNPs and InDels among the ten jujube genome assemblies were annotated using ANNOVAR (Wang et al., 2010). For gene-based pan-genome comparison, we used OrthoFinder (v2.5.4) (Emms and Kelly, 2019) to cluster the orthologous genes and categorized them into three groups: core (present in all seven assembly), private (unique to one assembly), and dispensable (present in two to six assemblies). Structural variations between 'Huizao' and 'Dongzao' were determined using Assemblytics and SyRI (v1.6.4) using 'Dongzao' as the reference (Goel et al., 2019; Li, 2018; Nattestad and Schatz, 2016). The visualization of structural variants was performed with Plotsr (Goel and Schneeberger, 2022).

**Divergence time estimation and gene family analysis**

The genome assemblies of ten eudicot species, including four jujube varieties with T2T genome assemblies (i.e., 'Huizao', 'Dongzao', 'Junzao', amd 'Suanzao'), *Rhamnella rubrinervis*, *Prunus dulcis*, *Prunus persica*, *Malus domestica*, *Arabidopsis thaliana* and *Vitis vinifera* were used for the analyses. The single-copy orthologous sequences determined by OrthoFinder (v2.5.4) (Emms and Kelly, 2019) were aligned using MAFFT (v7.310) (Katoh and Standley, 2013), and concatenated for the phylogenetic tree construction using RAxML (v8.2.12) (Stamatakis, 2014) with 1000 bootstraps. The divergence times were estimated using calibration points from the TimeTree website (timetree.org) with MCMCTree (v4.5) (Puttick, 2019). The expanded and contracted gene families were identified based on the OrthoFinder derived orthogroups and species tree results with CAFE (v5.0) (Mendes et al., 2021). GO enrichment analysis of significantly expanded and contracted gene sets were performed using ClusterProfiler (v4.8.3) (Yu et al., 2012).

JCVI pipeline (Tang et al., 2008) was used to find syntenic blocks among the jujube varieties with T2T assemblies. For each syntenic gene pair, synonymous substitutions per synonymous site (Ks) were determined with the gamma-NG method with ParaAT (Zhang et al., 2012). Protein sequences with GUB_WAK_bind domain in 'Huizao' and 'Dongzao' were filtered with pfamscan based on HMMER suite and blastp searching and verified by the existence of PF13947. Multiple alignments of all the hits were performed using ClustalW (Thompson et al., 1994); the Maximum Likelihood phylogenetic tree was constructed using the MEGA program (v5.2) with 1000 bootstrap replicates (Tamura et al., 2011). The homologous modeling of WAKs was constructed with SWISS-MODEL (https://swissmodel.expasy.org/) (Waterhouse et al., 2018).

**Analysis of ultramicroscopic structure of pulp cells of 'Huizao' and 'Dongzao'**

For ultra-structural observation, the freshly collected fruits of 'Huizao' and 'Dongzao' in five development stages were cut into blocks less than 1 mm^3^ in the TEM fixative and transferred into an EP tube with fresh TEM fixative at 4℃ for preservation and transportation. And then the tissues were washed using 0.1 M PB (pH 7.4) for 3 times (15 minutes each). Tissues avoid light post fixed with 1% OsO_4_ in 0.1 M PB (pH 7.4) for 2 hours at room temperature. After remove OsO_4_, the tissues are rinsed in 0.1 M PB (pH 7.4) for 3 times (15 minutes Each). Then the tissues were dehydrated at room temperature as followed: 30% ethanol for 20 minutes; 50% ethanol for 20 minutes; 70% ethanol for 20 minutes; 80% ethanol for 20 minutes; 95% ethanol for 20 minutes; two changes of 100% ethanol for 20 minutes; finally, two changes of acetone for 15 minutes. Resin penetration and embedding as follows: Acetone:EMBed 812=1:1 for 2-4 hours at 37℃; Acetone:EMBed 812=1:2 overnight at 37℃; pure EMBed 812 for 5-8 hours at 37℃. The embedding models with resin and samples were moved into 65℃ oven to polymerize for more than 48 hours. And then the resin blocks were taken out from the embedding models for standby application at room temperature. The resin blocks were cut to 60-80 nm thin on the ultra-microtome, and the tissues were fished out onto the 150 meshes cuprum grids with formvar film. 2% uranium acetate saturated alcohol solution avoid light staining for 8 min, rinsed in 70% ethanol for 3 times and then rinsed in ultra-pure water for 3 times. 2.6% lead citrate avoid CO_2_ staining for 8 minutes, and then rinsed with ultra-pure water for 3 times. After drying with filter paper, the grids were placed in a grid box and air-dried overnight at room temperature. Finally, samples were observed under TEM, and images were acquired.

**Measurements of physiological indices during jujube fruits development**

The fruits of 'Huizao' and 'Dongzao' during five developmental stages were collected and used for fruit texture-related physiological characteristics. After the outer peel was removed, three points were evenly selected to measure fruit firmness with a hand-held firmness instrument. The pectin content of the cell wall was measured with G0717W48 kit produced by Suzhou Gris Biotechnology Co., LTD. The prepared paraffin sections of fruits were observed and photographed under optical microscope (DM2700) and transmission electron microscope (TecnaiG220TWIN) with cell thickness measured with Image J (https://imagej.en.softonic.com/) software with three replicates.

**Transcriptome analysis**

The sequenced RNA-seq reads from five fruit developmental stages of 'Huizao' and 'Dongzao' were qualified with Trimmomatic (v0.39) (Bolger et al., 2014). Clean reads were aligned to the assembled 'Huizao' genome using HISAT (v2.2.1) and StringTie (v2.2.0) (Pertea et al., 2016). Gene expression levels were measured using the fragments per kb per million reads (FPKM). The differentially expressed genes were determined with edgeR (Baldoni et al., 2024)

**Baldoni, P.L., Chen, Y., Hediyeh-Zadeh, S., Liao, Y., Dong, X., Ritchie, M.E., Shi, W., and Smyth, G.K.** (2024). Dividing out quantification uncertainty allows efficient assessment of differential transcript expression with edgeR. Nucleic Acids Res **52:** e13.

**Benson, G.** (1999). Tandem repeats finder: a program to analyze DNA sequences. Nucleic Acids Research **27:** 573-580.

**Bolger, A.M., Lohse, M., and Usadel, B.** (2014). Trimmomatic: a flexible trimmer for Illumina sequence data. Bioinformatics (Oxford, England) **30:** 2114-2120.

**Brown, M., González De la Rosa, P.M., and Mark, B.** (2023). A Telomere Identification Toolkit. Zenodo.

**Burge, C., and Karlin, S.** (1997). Prediction of complete gene structures in human genomic DNA. J Mol Biol **268:** 78-94.

**Cantarel, B.L., Korf, I., Robb, S.M.C., Parra, G., Ross, E., Moore, B., Holt, C., Alvarado, A.S., and Yandell, M.** (2008). MAKER: An easy-to-use annotation pipeline designed for emerging model organism genomes. Genome research **18:** 188-196.

**Chan, P.P., and Lowe, T.M.** (2019). tRNAscan-SE: Searching for tRNA Genes in Genomic Sequences. Methods Mol Biol **1962:** 1-14.

**Chen, J., Wang, Z., Tan, K., Huang, W., Shi, J., Li, T., Hu, J., Wang, K., Wang, C., Xin, B., Zhao, H., Song, W., Hufford, M.B., Schnable, J.C., Jin, W., and Lai, J.** (2023). A complete telomere-to-telomere assembly of the maize genome. Nature genetics **55:** 1221-1231.

**Cheng, H., Concepcion, G.T., Feng, X., Zhang, H., and Li, H.** (2021). Haplotype-resolved de novo assembly using phased assembly graphs with hifiasm. Nat Methods **18:** 170-175.

**Dudchenko, O., Batra, S.S., Omer, A.D., Nyquist, S.K., Hoeger, M., Durand, N.C., Shamim, M.S., Machol, I., Lander, E.S., Aiden, A.P., and Aiden, E.L.** (2017). De novo assembly of the Aedes aegypti genome using Hi-C yields chromosome-length scaffolds. Science **356:** 92-95.

**Durand, N.C., Shamim, M.S., Machol, I., Rao, S.S., Huntley, M.H., Lander, E.S., and Aiden, E.L.** (2016). Juicer Provides a One-Click System for Analyzing Loop-Resolution Hi-C Experiments. Cell Syst **3:** 95-98.

**Emms, D.M., and Kelly, S.** (2019). OrthoFinder: phylogenetic orthology inference for comparative genomics. Genome Biol **20:** 238.

**Goel, M., and Schneeberger, K.** (2022). plotsr: visualizing structural similarities and rearrangements between multiple genomes. Bioinformatics (Oxford, England) **38:** 2922-2926.

**Goel, M., Sun, H., Jiao, W.B., and Schneeberger, K.** (2019). SyRI: finding genomic rearrangements and local sequence differences from whole-genome assemblies. Genome Biol **20:** 277.

**Guarracino, A., Heumos, S., Nahnsen, S., Prins, P., and Garrison, E.** (2022). ODGI: understanding pangenome graphs. Bioinformatics (Oxford, England) **38:** 3319-3326.

**Guo, M., Lian, Q., Mei, Y., Yang, W., Zhao, S., Zhang, S., Xing, X., Zhang, H., Gao, K., He, W., Wang, Z., Wang, H., Zhou, J., Cheng, L., Bao, Z., Huang, S., Yan, J., and Zhao, X.** (2024). Analyzes of pan-genome and resequencing atlas unveil the genetic basis of jujube domestication. Nat Commun **15:** 9320.

**Hickey, G., Monlong, J., Ebler, J., Novak, A.M., Eizenga, J.M., Gao, Y., Human Pangenome Reference, C., Marschall, T., Li, H., and Paten, B.** (2024). Pangenome graph construction from genome alignments with Minigraph-Cactus. Nat Biotechnol **42:** 663-673.

**Jain, C., Rhie, A., Zhang, H., Chu, C., Walenz, B.P., Koren, S., and Phillippy, A.M.** (2020). Weighted minimizer sampling improves long read mapping. Bioinformatics (Oxford, England) **36:** i111-i118.

**Jung, Y., and Han, D.** (2022). BWA-MEME: BWA-MEM emulated with a machine learning approach. Bioinformatics (Oxford, England) **38:** 2404-2413.

**Katoh, K., and Standley, D.M.** (2013). MAFFT multiple sequence alignment software version 7: improvements in performance and usability. Molecular biology and evolution **30:** 772-780.

**Li, H.** (2018). Minimap2: pairwise alignment for nucleotide sequences. Bioinformatics (Oxford, England) **34:** 3094-3100.

**Li, K., Chen, R., Abudoukayoumu, A., Wei, Q., Ma, Z., Wang, Z., Hao, Q., and Huang, J.** (2024). Haplotype-resolved T2T reference genomes for wild and domesticated accessions shed new insights into the domestication of jujube. Horticulture research **11:** uhae071.

**Majoros, W.H., Pertea, M., and Salzberg, S.L.** (2004). TigrScan and GlimmerHMM:: two open source eukaryotic gene-finders. Bioinformatics (Oxford, England) **20:** 2878-2879.

**Mendes, F.K., Vanderpool, D., Fulton, B., and Hahn, M.W.** (2021). CAFE 5 models variation in evolutionary rates among gene families. Bioinformatics (Oxford, England) **36:** 5516-5518.

**Murray, M.G., and Thompson, W.F.** (1980). Rapid isolation of high molecular weight plant DNA. Nucleic Acids Res **8:** 4321-4325.

**Nattestad, M., and Schatz, M.C.** (2016). Assemblytics: a web analytics tool for the detection of variants from an assembly. Bioinformatics (Oxford, England) **32:** 3021-3023.

**Nawrocki, E.P., and Eddy, S.R.** (2013). Infernal 1.1: 100-fold faster RNA homology searches. Bioinformatics (Oxford, England) **29:** 2933-2935.

**Ou, S., and Jiang, N.** (2018). LTR_retriever: A Highly Accurate and Sensitive Program for Identification of Long Terminal Repeat Retrotransposons. Plant physiology **176:** 1410-1422.

**Pertea, M., Kim, D., Pertea, G.M., Leek, J.T., and Salzberg, S.L.** (2016). Transcript-level expression analysis of RNA-seq experiments with HISAT, StringTie and Ballgown. Nat Protoc **11:** 1650-1667.

**Puttick, M.N.** (2019). MCMCtreeR: functions to prepare MCMCtree analyses and visualize posterior ages on trees. Bioinformatics (Oxford, England) **35:** 5321-5322.

**Simao, F.A., Waterhouse, R.M., Ioannidis, P., Kriventseva, E.V., and Zdobnov, E.M.** (2015). BUSCO: assessing genome assembly and annotation completeness with single-copy orthologs. Bioinformatics (Oxford, England) **31:** 3210-3212.

**Song, J.M., Xie, W.Z., Wang, S., Guo, Y.X., Koo, D.H., Kudrna, D., Gong, C., Huang, Y., Feng, J.W., Zhang, W., Zhou, Y., Zuccolo, A., Long, E., Lee, S., Talag, J., Zhou, R., Zhu, X.T., Yuan, D., Udall, J., Xie, W., Wing, R.A., Zhang, Q., Poland, J., Zhang, J., and Chen, L.L.** (2021). Two gap-free reference genomes and a global view of the centromere architecture in rice. Mol Plant **14:** 1757-1767.

**Stamatakis, A.** (2014). RAxML version 8: a tool for phylogenetic analysis and post-analysis of large phylogenies. Bioinformatics (Oxford, England) **30:** 1312-1313.

**Stanke, M., Diekhans, M., Baertsch, R., and Haussler, D.** (2008). Using native and syntenically mapped cDNA alignments to improve de novo gene finding. Bioinformatics (Oxford, England) **24:** 637-644.

**Tamura, K., Peterson, D., Peterson, N., Stecher, G., Nei, M., and Kumar, S.** (2011). MEGA5: molecular evolutionary genetics analysis using maximum likelihood, evolutionary distance, and maximum parsimony methods. Molecular biology and evolution **28:** 2731-2739.

**Tang, H., Bowers, J.E., Wang, X., Ming, R., Alam, M., and Paterson, A.H.** (2008). Synteny and collinearity in plant genomes. Science **320:** 486-488.

**Thompson, J.D., Higgins, D.G., and Gibson, T.J.** (1994). CLUSTAL W: improving the sensitivity of progressive multiple sequence alignment through sequence weighting, position-specific gap penalties and weight matrix choice. Nucleic Acids Res **22:** 4673-4680.

**Wang, K., Li, M., and Hakonarson, H.** (2010). ANNOVAR: functional annotation of genetic variants from high-throughput sequencing data. Nucleic Acids Res **38:** e164.

**Waterhouse, A., Bertoni, M., Bienert, S., Studer, G., Tauriello, G., Gumienny, R., Heer, F.T., de Beer, T.A.P., Rempfer, C., Bordoli, L., Lepore, R., and Schwede, T.** (2018). SWISS-MODEL: homology modelling of protein structures and complexes. Nucleic Acids Res **46:** W296-W303.

**Wei, T., Li, H., Huang, X., and Yang, P.** (2024). Chromosome-level genome assembly of two cultivated Jujubes. Sci Data **11:** 1144.

**Yang, M., Han, L., Zhang, S., Dai, L., Li, B., Han, S., Zhao, J., Liu, P., Zhao, Z., and Liu, M.** (2023). Insights into the evolution and spatial chromosome architecture of jujube from an updated gapless genome assembly. Plant Commun **4:** 100662.

**Yu, G., Wang, L.G., Han, Y., and He, Q.Y.** (2012). clusterProfiler: an R package for comparing biological themes among gene clusters. OMICS **16:** 284-287.

**Zdobnov, E.M., and Apweiler, R.** (2001). InterProScan - an integration platform for the signature-recognition methods in InterPro. Bioinformatics (Oxford, England) **17:** 847-848.

**Zhang, Z., Xiao, J., Wu, J., Zhang, H., Liu, G., Wang, X., and Dai, L.** (2012). ParaAT: a parallel tool for constructing multiple protein-coding DNA alignments. Biochem Biophys Res Commun **419:** 779-781.
