# Supplementary material for: The T2T genome assembly of Ziziphus jujuba ‘Huizao’ and pan-genome analyses provide insights into fruit texture diversity in jujube
Source: Mol Hortic. 2026 Jun 5;6:42. doi: 10.1186/s43897-025-00228-1 (PMC13238111; doi:10.1186/s43897-025-00228-1)
Supplement: Supplementary file 3 — Supplementary Material 3. [file 43897_2025_228_MOESM3_ESM.docx]

**Table S1.** Summary of genome sequencing data of *Ziziphus jujuba* 'Huizao'*.*

| **Category** | **Illumina** | **HiFi** | **ONT** | **HIC** |
| --- | --- | --- | --- | --- |
| **Read number** | 207,622,077 (pairs) | 1,937,720 | 365,348 | 167,268,072(pairs) |
| **Total length (Gb)** | 62.29 | 30.42 | 36.63 | 50.18 |
| **Depth** | 149× | 72× | 87× | 118× |
| **Average Read length (bp)** | 150 | 15,697 | 100,270 | 150 |

**Table S2**. Chromosome statics of the T2T genome assemblies of 'Huizao'.

|  | **HZ_hapA** | | **HZ_hapB** | |
| --- | --- | --- | --- | --- |
| **Chromosome** | Gap Number | Chromosome Length (bp) | Gap Number | Chromosome Length (bp) |
| **Chr01** | 0 | 48,139,315 | 0 | 47,438,248 |
| **Chr02** | 0 | 29,189,661 | 0 | 28,562,776 |
| **Chr03** | 0 | 30,858,206 | 0 | 30,403,847 |
| **Chr04** | 0 | 32,694,372 | 0 | 32,874,511 |
| **Chr05** | 0 | 34,403,722 | 0 | 32,823,326 |
| **Chr06** | 0 | 32,716,953 | 0 | 32,994,633 |
| **Chr07** | 0 | 34,095,478 | 0 | 32,252,923 |
| **Chr08** | 0 | 29,024,745 | 0 | 28,633,016 |
| **Chr09** | 0 | 30,412,833 | 0 | 30,739,056 |
| **Chr10** | 0 | 26,418,417 | 0 | 25,533,727 |
| **Chr11** | 0 | 28,458,416 | 0 | 31,253,916 |
| **Chr12** | 0 | 28,533,132 | 0 | 28,001,135 |

**Table S3**. Genome assembly statistics of *Ziziphus jujuba* 'Huizao' and its comparison with three other T2T jujube genomes.

| Feature | *Z. jujuba* 'Huizao' (HZ) | *Z. jujuba* 'Dongzao' (DZ) | *Z. jujuba* 'Junzao' (JZ) | *Z. jujuba* var. *spinosa* 'Suanzao' (SZ) |
| --- | --- | --- | --- | --- |
| Size of assembly (bp) | 384,945,250 | 393,332,932 | 385,801,156 | 375,153,944 |
| Anchored rates (%) | 100% | 100% | 100% | 100% |
| Number of chromosomes (2n) | 2×12 | 2×12 | 2×12 | 2×12 |
| contig N50 (bp) | 30,858,206 | 32,986,920 | 31,079,923 | 30,843,711 |
| BUSCO completeness of genome (%) | 98.30% | 98.50% | 98.70% | 98.20% |
| LTR assembly index (LAI) | 20.46 | 17.89 | 16.42 | 17.89 |
| Repeat sequence (Mb) | 193,76 | 220.88 | 202.43 | 209.57 |
| Number of protein-coding genes | 30,369 | 31,115 | 27,651 | 26,658 |

**Table S4.** The genome assembly and annotation completeness of the 'Huizao' genome assemblies assessed by BUSCO.

|  | **HZ_hapA** | **HZ_hapB** |
| --- | --- | --- |
| **Complete BUSCOs** | 98.20% | 98.30% |
| **Complete and single-copy BUSCOs** | 97.00% | 97.30% |
| **Complete and duplicated BUSCOs** | 1.20% | 1.00% |
| **Fragmented BUSCOs** | 0.90% | 0.90% |
| **Missing BUSCOs** | 0.90% | 0.80% |

**Table S5**. Statistics of centromere and telomere distribution on each chromosome of the 'Huizao' genome.

|  | **Centromere** | | | | | | |  |
| --- | --- | --- | --- | --- | --- | --- | --- | --- |
|  | Chromosome | Chr_length | | Centro_start | Centro_end | Centro_length |  |  |
| **HZ_hapA** | Chr01 | 48,139,315 | | 6,480,000 | 6,710,000 | 230,001 |  |  |
|  | Chr02 | 29,189,661 | | 5,210,000 | 5,560,000 | 350,001 |  |  |
|  | Chr03 | 30,858,206 | | 6,250,000 | 6,280,000 | 30,001 |  |  |
|  | Chr04 | 32,694,372 | | 15,600,000 | 15,700,000 | 100,001 |  |  |
|  | Chr05 | 34,403,722 | | 17,370,000 | 17,990,000 | 620,001 |  |  |
|  | Chr06 | 32,716,953 | | 11,950,000 | 12,840,000 | 890,001 |  |  |
|  | Chr07 | 34,095,478 | | 18,320,000 | 18,360,000 | 40,001 |  |  |
|  | Chr08 | 29,024,745 | | 13,020,000 | 13,240,000 | 220,001 |  |  |
|  | Chr09 | 30,412,833 | | 16,040,000 | 16,550,000 | 510,001 |  |  |
|  | Chr10 | 26,418,417 | | 7,880,000 | 7,950,000 | 70,001 |  |  |
|  | Chr11 | 28,458,416 | | 15,790,000 | 16,560,000 | 770,001 |  |  |
|  | Chr12 | 28,533,132 | | 12,840,000 | 12,870,000 | 30,001 |  |  |
| **HZ_hapB** | Chr01 | 47,438,248 | | 5,770,000 | 5,800,000 | 30,001 |  |  |
|  | Chr02 | 28,562,776 | | 2,720,000 | 3,000,000 | 280,001 |  |  |
|  | Chr03 | 30,403,847 | | - | - | - |  |  |
|  | Chr04 | 32,874,511 | | 4,620,000 | 4,840,000 | 220,001 |  |  |
|  | Chr05 | 32,823,326 | | 16,920,000 | 17,390,000 | 470,001 |  |  |
|  | Chr06 | 32,994,633 | | 11,460,000 | 11,530,000 | 70,001 |  |  |
|  | Chr07 | 32,252,923 | | 3,600,000 | 3,720,000 | 120,001 |  |  |
|  | Chr08 | 28,633,016 | | 14,360,000 | 14,410,000 | 50,001 |  |  |
|  | Chr09 | 30,739,056 | | 29,700,000 | 29,830,000 | 130,001 |  |  |
|  | Chr10 | 25,533,727 | | 7,750,000 | 7,850,000 | 100,001 |  |  |
|  | Chr11 | 31,253,916 | | 19,310,000 | 19,460,000 | 150,001 |  |  |
|  | Chr12 | 28,001,135 | | 13,620,000 | 13,720,000 | 100,001 |  |  |
|  | **Telomere** | | | | | | |  |
| **HZ_hapA** | Chromosome | Left_start | Left_end | | Left_length | Right_start | Right_end | Right_length |
|  | Chr01 | 301 | 14,242 | | 13,942 | 48,128,215 | 48,139,220 | 11,006 |
|  | Chr02 | 49 | 7,075 | | 7,027 | 29,180,665 | 29,189,564 | 8,900 |
|  | Chr03 | 27 | 939 | | 913 | 30,856,230 | 30,858,204 | 1,975 |
|  | Chr04 | 4 | 7,499 | | 7,496 | 32,680,445 | 32,694,372 | 13,928 |
|  | Chr05 | 173 | 13,297 | | 13,125 | 34,395,329 | 34,403,523 | 8,195 |
|  | Chr06 | 11 | 6,845 | | 6,835 | 32,711,456 | 32,716,896 | 5,441 |
|  | Chr07 | 80 | 6,429 | | 6,350 | 34,089,647 | 34,095,463 | 5,817 |
|  | Chr08 | 119 | 9,166 | | 9,048 | 29,023,807 | 29,024,719 | 913 |
|  | Chr09 | 30 | 5,535 | | 5,506 | 30,391,147 | 30,412,778 | 21,632 |
|  | Chr10 | 97 | 5,590 | | 5,494 | 26,415,976 | 26,418,404 | 2,429 |
|  | Chr11 | 28 | 6,600 | | 6,573 | 28,453,358 | 28,458,399 | 5,042 |
|  | Chr12 | 15 | 7,188 | | 7,174 | 28,520,970 | 28,532,994 | 12,025 |
| **HZ_hapB** | Chr01 | 251 | 5,002 | | 4,752 | 47,432,249 | 47,438,228 | 5,980 |
|  | Chr02 | 40 | 7,525 | | 7,486 | 28,550,583 | 28,562,647 | 12,065 |
|  | Chr03 | 6 | 3,430 | | 3,425 | 30,401,992 | 30,403,845 | 1,854 |
|  | Chr04 | 63 | 7,046 | | 6,984 | 32,873,956 | 32,874,356 | 401 |
|  | Chr05 | 1 | 8,189 | | 8,189 | 32,814,203 | 32,823,127 | 8,925 |
|  | Chr06 | - | - | | - | 32,989,522 | 32,994,629 | 5,108 |
|  | Chr07 | 52 | 12,995 | | 12,944 | 32,244,608 | 32,252,827 | 8,220 |
|  | Chr08 | 45 | 3,932 | | 3,888 | 28,631,144 | 28,632,935 | 1,792 |
|  | Chr09 | 195 | 5,571 | | 5,377 | 30,729,563 | 30,739,047 | 9,485 |
|  | Chr10 | 75 | 11,502 | | 11,428 | 25,522,945 | 25,533,676 | 10,732 |
|  | Chr11 | 119 | 16,267 | | 16,149 | 31,245,018 | 31,253,900 | 8,883 |
|  | Chr12 | 180 | 3,610 | | 3,431 | 27,994,344 | 28,001,104 | 6,761 |

**Table S6**. Summary of repeat elements identified in the 'Huizao' genome.

| **HZ_hapA** | | | | **HZ_hapB** | | | |
| --- | --- | --- | --- | --- | --- | --- | --- |
| **Classification** | **Number** | **Length (bp)** | **Percentage in the genome** | **Classification** | **Number** | **Length (bp)** | **Percentage in the genome** |
| **DNA** | 14,299 | 3,503,699 | 0.91% | DNA | 20748 | 7,691,944 | 2.02% |
| **DNA/CMC-EnSpm** | 3,728 | 1,824,694 | 0.47% | DNA/CMC-EnSpm | 4761 | 3,511,092 | 0.92% |
| **DNA/En-Spm** | 31 | 24,302 | 0.01% | DNA/En-Spm | 433 | 28,060 | 0.01% |
| **DNA/Harbinger** | 1 | 161 | 0.00% | DNA/Harbinger | 372 | 164,542 | 0.04% |
| **DNA/MULE-MuDR** | 4,131 | 2,707,959 | 0.70% | DNA/MULE-MuDR | 5170 | 3,345,831 | 0.88% |
| **DNA/MuDR** | 2,225 | 2,351,230 | 0.61% | DNA/MuDR | 2125 | 1,933,167 | 0.51% |
| **DNA/PIF-Harbinger** | 1,022 | 874,294 | 0.23% | DNA/PIF-Harbinger | 1058 | 833,887 | 0.22% |
| **DNA/TcMar-ISRm11** | 87 | 51,108 | 0.01% | DNA/Zisupton | 143 | 52,736 | 0.01% |
| **DNA/hAT-Ac** | 1,340 | 908,822 | 0.24% | DNA/hAT-Ac | 858 | 578,924 | 0.15% |
| **DNA/hAT-Tag1** | 2,631 | 1,302,577 | 0.34% | DNA/hAT-Tag1 | 2381 | 1,224,561 | 0.32% |
| **DNA/hAT-Tip100** | 699 | 248,669 | 0.07% | DNA/hAT-Tip100 | 2008 | 495,798 | 0.13% |
| **DNAauto/PILETAA** | 133 | 74,977 | 0.02% |  |  |  |  |
| **LINE/L1** | 6,725 | 2,919,774 | 0.76% | LINE/L1 | 4169 | 2,626,578 | 0.69% |
| **LTR** | 45,155 | 22,207,867 | 5.77% | LTR | 41461 | 23,073,391 | 6.05% |
| **LTR/Caulimovirus** | 598 | 296,981 | 0.08% | LTR/Caulimovirus | 1590 | 500,462 | 0.13% |
| **LTR/Copia** | 63,369 | 31,139,515 | 8.09% | LTR/Copia | 61211 | 31,647,679 | 8.30% |
| **LTR/Gypsy** | 66065 | 59,379,661 | 15.43% |  |  |  |  |
| **LTR/Pao** | 204 | 53,137 | 0.01% | LTR/Gypsy | 59686 | 52,255,465 | 13.70% |
| **RC/Helitron** | 7024 | 3,408,016 | 0.89% | RC/Helitron | 4411 | 2,043,822 | 0.54% |
| **Unknown** | 230055 | 60,463,381 | 15.71% | Unknown | 224960 | 58,482,754 | 15.33% |
| **Simple_repeat** | 316 | 15738 | 0.00% | Simple_repeat | 77 | 2,521 | 0.00% |
| **Total** | 449838 | 1.94E+08 | 50.33% | Total | 437622 | 1.9E+08 | 49.93% |

**Table S7**. Summary of genes identified in the 'Huizao' genome.

|  | **HZ_hapA** | **HZ_hapB** |
| --- | --- | --- |
| **Statistics** | Number & Length | Number & Length |
| **Number of genes** | 30,369 | 30,005 |
| **Total genic length** | 1.14E+08 | 1.2E+08 |
| **Mean gene length** | 3,752 | 4,003 |
| **Number of transcripts** | 35,911 | 35,405 |
| **Transcripts per gene** | 1.2 | 1.2 |
| **Total transcript length** | 65,986,207 | 65,808,732 |
| **Mean transcript length** | 1,837 | 1,858 |
| **Number of exons** | 210,431 | 210,633 |
| **Exons per transcript** | 5.9 | 5.9 |
| **Mean exon length** | 313 | 312 |
| **Number of coding exons** | 199,934 | 200,471 |
| **Number of introns** | 174,520 | 175,228 |
| **Mean intron length** | 431 | 462 |
| **Total cds length** | 50,742,795 | 50,785,925 |
| **Mean CDS length** | 1,413 | 1,434 |

**Table S8.** Annotation statistics of predicted protein-coding genes in the 'Huizao' genome.

| **Category** | | **HZ_hapA** | | | **HZ_hapB** | |
| --- | --- | --- | --- | --- | --- | --- |
|  | **Number** | | **Percentage** | **Number** | | **Percentage** |
| Total genes | 30,369 | | 100.00% | 30,005 | | 100.00% |
| NCBI nr | 29,214 | | 96.20% | 28,878 | | 96.24% |
| Swiss-Prot | 21,679 | | 71.39% | 21,518 | | 71.71% |
| eggNOG | 26,938 | | 88.70% | 26,582 | | 88.59% |
| KEGG | 11,636 | | 38.32% | 11,491 | | 38.30% |
| GO | 13,612 | | 44.82% | 13,434 | | 44.77% |

**Table S9. S**tatistics of graph-based jujube pan-genome.

| **Chromosome** | **Types** | **Size(bp)** |
| --- | --- | --- |
| **Chr01** | nodes | 3741946 |
| **Chr01** | edges | 5115601 |
| **Chr01** | length | 67196119 |
| **Chr02** | nodes | 2792216 |
| **Chr02** | edges | 3811784 |
| **Chr02** | length | 53012138 |
| **Chr03** | nodes | 2872007 |
| **Chr03** | edges | 3921850 |
| **Chr03** | length | 54224747 |
| **Chr04** | nodes | 2777963 |
| **Chr04** | edges | 3801642 |
| **Chr04** | length | 47956551 |
| **Chr05** | nodes | 2741215 |
| **Chr05** | edges | 3751875 |
| **Chr05** | length | 44928508 |
| **Chr06** | nodes | 2274029 |
| **Chr06** | edges | 3108005 |
| **Chr06** | length | 42432455 |
| **Chr07** | nodes | 2269692 |
| **Chr07** | edges | 3110612 |
| **Chr07** | length | 41249023 |
| **Chr08** | nodes | 2304238 |
| **Chr08** | edges | 3153846 |
| **Chr08** | length | 44310193 |
| **Chr09** | nodes | 2727190 |
| **Chr09** | edges | 3736424 |
| **Chr09** | length | 45333679 |
| **Chr10** | nodes | 2448931 |
| **Chr10** | edges | 3353332 |
| **Chr10** | length | 42275220 |
| **Chr11** | nodes | 1850687 |
| **Chr11** | edges | 2535077 |
| **Chr11** | length | 36413542 |
| **Chr12** | nodes | 2057187 |
| **Chr12** | edges | 2813451 |
| **Chr12** | length | 36948758 |

**Table S10**. Summary of structural and sequence variation between the 'Huizao' and 'Dongzao' genome assemblies.

| **Structural annotations** | |  |  |
| --- | --- | --- | --- |
| Variation_type | Count | Length_ref | Length_qry |
| Syntenic regions | 86 | 373,990,343 | 375,424,098 |
| Inversions | 17 | 1,016,959 | 1,172,941 |
| Translocations | 50 | 1,045,917 | 1,136,532 |
| Duplications (reference) | 21 | 142,486 | - |
| Duplications (query) | 187 | - | 2,306,155 |
| Not aligned (reference) | 152 | 9,679,264 | - |
| Not aligned (query) | 287 | - | 13,565,606 |
| **Sequence annotations** | |  |  |
| Variation_type | Count | Length_ref | Length_qry |
| SNPs | 1,335,813 | 1,335,813 | 1,335,813 |
| Insertions | 145,317 | - | 2,450,935 |
| Deletions | 163,612 | 2,764,461 | - |
| Copygains | 34 | - | 79,470 |
| Copylosses | 44 | 67,449 | - |
| Highly diverged | 9,557 | 89,422,001 | 91,399,474 |
| Tandem repeats | 10 | 21,351 | 19,420 |
