# Supplementary material for: The T2T genome assembly of Ziziphus jujuba ‘Huizao’ and pan-genome analyses provide insights into fruit texture diversity in jujube
Source: Mol Hortic. 2026 Jun 5;6:42. doi: 10.1186/s43897-025-00228-1 (PMC13238111; doi:10.1186/s43897-025-00228-1)
Supplement: Supplementary file 1 — Supplementary Material 1. [file 43897_2025_228_MOESM1_ESM.docx]

Figure S1. Assembly of *Z. jujuba* 'Huizao' T2T genome. (a), (b) Photograph of the tree and fruit of *Z. jujuba* 'Huizao'. (c), (d) Hi-C contact maps of the two *Z. jujuba* 'Huizao' subgenome assemblies. Strong and weak interactions are indicated in dark red and white color, respectively.

Figure S2. Pan-genome graph of jujube.

Figure S3. Evolutionary analyses of *Z. jujuba* 'Huizao'. (a) The phylogenetic tree of ten species based on 1,718 single-copy orthologous genes. Estimated divergence times are indicated at the nodes. Numbers of gene family expansion and contraction are shown above the nodes and taxa, respectively; green and red colors indicate the number of expanded and contracted gene families, respectively. (b) Distribution of Ks for pairs of paralogs/orthologs. (c) Venn diagram of shared and specific orthologous gene families among 'Huizao', 'Dongzao', 'Junzao', and 'Suanzao'. (d), (e) GO enrichment analyses of expanded and specific gene families in the 'Huizao' genome.

Figure S4. Comparative genomic analyses. (a) Phylogenetic analysis of protein sequences with GUB_WAK_bind domain in the 'Huizao' and 'Dongzao' genomes; clades (clade-a, clade-b, clade-c) with expanded gene copies in 'Huizao' are highlighted with green colors. (b) Chromosome distribution of clade-b and clade-c; the collinear links of expanded GUB_WAK_bind genes are highlighted with green colors, and the collinear links of other genes are shown with grey colors. Expanded GUB_WAK_bind genes in 'Huizao' are labeled with green stars; the adjacent transposons and chaperone genes are labeled with red and blue stars, respectively. (c) Representative domain distribution of GUB_WAK_bind contained protein groups identified in a. (d) Protein tertiary structure of WAK predicted by homologous modeling.

Figure S5. Circos phylogenetic tree of protein sequences with GUB_WAK_bind domain identified in the 'Huizao' and 'Dongzao' genomes. Proteins from *Arabidopsis thaliana* are highlighted by red color, expanded copies in the 'Huizao' genome are highlighted with green color.

Figure S6. Rectangular phylogenetic tree of proteins with GUB_WAK_bind domain identified in the 'Huizao' and 'Dongzao' genomes. Proteins from *Arabidopsis thaliana* are highlighted by red color, expanded gene copies are highlighted with green color. Support rates are shown at the nodes.

Figure S7. Chromosome distribution of GUB_WAK_bind genes expanded in the Huizao genome. The collinear links of GUB_WAK_bind genes between 'Huizao' and 'Dongzao' are highlighted with green color, the collinear links of other genes are shown with grey color. Gene symbols were labeled with small fonts.

**Figure S8.** Size distribution of structural variations (SVs) between 'Huizao' and 'Dongzao' genomes.

Figure S9. Comparison of fruit development and gene expression between 'Huizao' and 'Dongzao'. (a) Differential expression of GUB_WAK_bind genes between 'Huizao' and 'Dongzao' across the five developmental stages. Group labels are indicated above each subplot. (b) Structural variation surrounding differentially expressed GUB_WAK_bind genes (e.g., Chr01.g01500), with HZ_hapA as the reference genome.

Figure S10. Ultrastructure of pulp cells at five developmental stages. From left to right and top to bottom are DZ (young, expansion, white ripening, half ripening, and full ripening) and HZ (young, expansion, white ripening, half ripening, and full ripening).

Figure S11. GO enrichment analysis of differentially expressed genes at young fruit stage between 'Huizao' and 'Dongzao'.

Figure S12. GO enrichment analysis of differentially expressed genes at expansion stage between 'Huizao' and 'Dongzao'.

Figure S13. GO enrichment analysis of differentially expressed genes at full ripening stage between 'Huizao' and 'Dongzao'.

Figure S14. Representative diagrams of differentially expressed GUB_WAK_bind genes (Chr04.g14064 and Chr05.g17426) with significant structural variation. The HZ_HapA genome assembly was used as the reference.
